# Supplementary material for: Sustained-input switches for transcription factors and microRNAs are central building blocks of eukaryotic gene circuits
Source: Genome Biol. 2013 Aug 23;14(8):R85. doi: 10.1186/gb-2013-14-8-r85 (PMC4054853; doi:10.1186/gb-2013-14-8-r85)
Supplement: Additional file 5 — HTML Browsable Motif Output. Zipped folder containing all WaRSwap and FANMOD motif output, viewable in a web browser. [file gb-2013-14-8-r85-S5.ZIP › HTML_browsable_motif_output/WaRSwap_all_tair9/sigs_WSWRSLDraw-2000_all.pvals.heatmaps.html/motif_id_14_011110001_tftype_all_upstream_-1000_0.html]

```
BG_MODEL = Rnew2
MOTIF_ID = 14_011110001
TF_TYPE = all
UPSTREAM = -1000_0


PVals
FN_0.2	FN_0.4	FN_0.6	FN_0.8
dg_60.genes	0.2	0.018	0.617	0.495
dg_70.genes	0.142	0.009	0.564	0.484
dg_80.genes	0.465	0.083	0.816	0.391

ZScores
FN_0.2	FN_0.4	FN_0.6	FN_0.8
dg_60.genes	0.863	2.664	-0.488	-0.645
dg_70.genes	1.095	3.003	-0.375	-0.654
dg_80.genes	-0.005	1.546	-0.91	-0.571

StDevs
FN_0.2	FN_0.4	FN_0.6	FN_0.8
dg_60.genes	653.765	238.786	58.823	7.941
dg_70.genes	635.185	223.851	55.357	7.414
dg_80.genes	517.091	187.932	47.231	6.268
```
